# PsychoPy—Psychophysics software in Python

**DOI:** 10.1016/j.jneumeth.2006.11.017

**Published:** 2007-05-15

**Authors:** Jonathan W. Peirce

**Affiliations:** Nottingham Visual Neuroscience, School of Psychology, University of Nottingham, Nottingham NG7 2RD, United Kingdom

**Keywords:** Psychophysics, Software, Stimulus presentation, Psychometric, Vision

## Abstract

The vast majority of studies into visual processing are conducted using computer display technology. The current paper describes a new free suite of software tools designed to make this task easier, using the latest advances in hardware and software. PsychoPy is a platform-independent experimental control system written in the Python interpreted language using entirely free libraries. PsychoPy scripts are designed to be extremely easy to read and write, while retaining complete power for the user to customize the stimuli and environment.

Tools are provided within the package to allow everything from stimulus presentation and response collection (from a wide range of devices) to simple data analysis such as psychometric function fitting. Most importantly, PsychoPy is highly extensible and the whole system can evolve via user contributions. If a user wants to add support for a particular stimulus, analysis or hardware device they can look at the code for existing examples, modify them and submit the modifications back into the package so that the whole community benefits.

## 1 Introduction

Since the 1980s computers and cathode-ray-tube displays (CRTs) have been used extensively, almost ubiquitously, in visual and cognitive neuroscience experiments. Despite the spatial and temporal limitations of the displays (Bach et al., 1997) and assorted other potential problems (Bach, 1997; Pelli, 1997a,b; Wolf and Deubel, 1997) the variety of stimuli that they can generate with relatively little effort has made them the stimulus presentation method of choice for most neuroscience laboratories.

This paper describes PsychoPy, a new suite of software tools to make it easier to build simple visual and auditory stimuli for neuroscience experiments. The goal of the project was to produce a package that was entirely free, as easy as possible to use, and based on relatively inexpensive (and preferably vendor-independent) hardware. The result is a set of tools built on top of the Python programming language that makes calls directly to OpenGL graphics libraries. These tools are fully platform-independent (for the major operating systems) and can interface freely and simply with an extremely wide range of additional hardware.

## 2 Methods and materials

### 2.1 Hardware

PsychoPy has been developed predominantly on the Microsoft Windows^®^ XP platform but has been extensively tested on Mac OS X (10.3 and 10.4) and has been used in experiments on both platforms. The necessary Python libraries on which it is based are also available on Linux and some users have reported success on that platform although it has received less complete testing as yet. The package is highly portable because it uses a minimal amount of compiled (e.g. C-based) code.

One of the minimum requirements for PsychoPy is a graphics card that supports OpenGL drivers and multitexturing. This includes almost every graphics card made by nVidia, ATI and Matrox since the late 1990s, although on the Microsoft platform the user may need to download additional drivers from the graphics card vendor rather than using the ones installed with Windows^®^. For experiments using a few simple stimuli (such as a pair of Gabor patches and a fixation point) basic versions of these cards or motherboards with built-in graphics processors are likely to suffice. For experiments that need to draw a large number of stimuli (such as random dot displays or global form patterns) a more powerful graphics card, a fast CPU, and plenty of memory can all result in performance gains.

### 2.2 Python

Many neuroscience labs around the world are using Matlab^®^ (The MathWorks Inc., Massachusetts, USA) for the generation of experimental stimuli via Psychtoolbox (Brainard, 1997; Pelli, 1997a,b) and for data analysis. This has the advantages of being a relatively platform-independent language with a fairly simple syntax and numerous high-level libraries. Matlab^®^ does have certain downsides however. It is expensive and, as a proprietary software solution, comes without source code which leaves the science community heavily reliant on its customer support services. This downside has been clearest in the company's unwillingness at times to support the Apple platform.

The goals in using Python are similar to those in using Matlab^®^. They are both high-level, extensible, interpreted languages, but there are several key differences. Python has a much cleaner syntax, making code easier to read and debug. It is completely open source and continuously developed on all platforms, each of which has its own strong user base. When bugs or incompatibilities are found they are generally very quickly fixed and, because the full source code is available, users can actually debug or change Python themselves (if they are sufficiently competent programmers). Another major advantage to the developer (rather than necessarily the user) is that Python has a large set of libraries already built, including a complete interface to OpenGL calls. These greatly reduce the need for platform-specific C-code. Indeed, PsychoPy is written almost entirely in native Python code.

The main downsides of Python, at least for the casual user, are that installation can require more effort. When a user installs Python for the purposes of neuroscience experiments they typically need to install around 10 auxiliary libraries to handle the functions such as data handling and plotting, stimulus drawing, hardware interfaces, etc., where many of these might have been included in a single Matlab^®^ installation. There is also, of course, a time investment in learning a new syntax. Speaking for myself, I certainly felt that the advantages of the improved syntax and absence of license fees warranted the investment of that time.

### 2.3 The use of OpenGL

Historically, interpreted languages such as Matlab^®^ and Python have not been fast enough to perform computations and generate stimuli on-the-fly in real time. As a result people have resorted to pre-computing stimulus movies or by manipulating the computer's color look-up table (CLUT) to create dynamic stimuli such as drifting gratings. Even compiled languages such as C/C++ were previously unable to generate moving stimuli such as a drifting Gabor patch (a sinusoidal grating drifting behind a Gaussian-enveloped window) in real-time. The problem is that for a patch with, say, 256 × 256 pixels the intensity value must be calculated for over 65,000 pixels in the stimulus on every frame and then the entire array has to be sent from the main computer memory to the frame buffer. Since most computers were too slow to perform the calculations and transmit the data to the graphics card within the requisite 10 ms, movies of these stimuli had to be pre-computed to perform the task.

Several advances have meant that this problem is no longer an issue. The central processing unit (CPU) obviously runs much faster than it used to, and the speed with which data is transferred to the graphics card memory is also vastly improved, but an even more important development for generation of these stimuli is that of hardware-accelerated graphics. Most graphics cards on standard personal computers now have independent processors (the graphics processing unit or GPU) which, through libraries such as OpenGL or Microsoft's DirectX^®^, are able to perform very fast matrix mathematical functions, without using the CPU or the data bus that connects it to the graphics card. PsychoPy uses this fact and preloads the graphics card with component patterns such as sinusoids and Gaussian envelopes at the start of the experiment. Then, when a stimulus is needed at a particular orientation, phase and position, the GPU is able to do the work of identifying how these components need to be combined (e.g. putting the sinusoid behind the Gaussian window, orienting it and calculating the changes needed in the frame buffer). This can generally be performed for several hundred stimuli in much less time than one computer frame and with very little impact on the CPU. As a result the CPU is left to handle other tasks such as communicating with hardware and waiting for events such as subject responses or MR scanner triggers. The drawing processes also require very few commands to be issued, which means that the overhead of using an interpreted language is diminished.

The concept can easily be extended to encompass second order stimuli such as contrast-modulated noise stimuli where three component matrices (a carrier, an envelope and a mask) are combined in one operation by the graphics card. Similarly, by using OpenGL's notion of alpha channels (transparency) we can trivially overlay multiple semi-transparent gratings to create stimuli such as plaids. As a result of these processes being so quick and executed with minimal input from the CPU, a large number of stimuli can be rendered, so that stimuli such as moving random dot displays can be generated in real-time and can even be generated using more complex elements like Gabors or difference-of-Gaussian patches.

## 3 Results

### 3.1 What PsychoPy provides

The primary functions of PsychoPy were designed to handle stimulus display and timing. They allow the user to generate a window (or full-screen presentation) and provide some basic stimuli to use within that window (e.g. random-dot-kinematograms, drifting grating stimuli, text, photographic images). In addition to the stimuli provided (or modifications of them), the user can generate entirely new stimuli by issuing OpenGL commands directly to the window, or combine pre-packaged stimuli with their own commands. In addition to the visual presentation, for which it was originally designed, PsychoPy is able to present stereo auditory stimuli using the computer sound card. Responses can easily be gathered via the keyboard and mouse, by standard devices such as joysticks, or by more elaborate hardware via the serial or parallel ports.

**Fig. 1:**
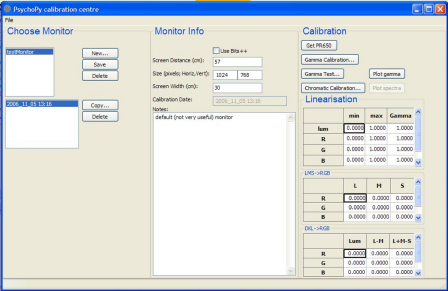
The MoniterCenter application provides the experimenter with a handy tool to provide information about their monitor to perform fully automated calibrations with a PR650, and to store information and notes from previous calibrations.

PsychoPy also provides a graphical-user-interface (GUI) application called MonitorCenter to manage calibration of monitors and store information from previous calibrations. The user simply inputs the dimensions of the monitor and its distance (using the GUI) allowing PsychoPy to convert units between various coordinate systems such as degrees of visual angle, centimeters or pixels. In addition MonitorCenter also stores gamma-correction parameters for the monitor, which are then applied automatically during experimental scripts. Furthermore, if a Spectrascan PR650 is connected to the serial port MonitorCenter can also perform a fully automated calibration at the touch of a button. This calibration will measure the luminance at a series of intensity levels and fit the optimum gamma-function to each gun. The PR650 will also measure the intensity spectrum for each gun to allow transformations between various color spaces. The result is that experimental scripts can simply request an image, for example, of a 2° width, with spatial frequency of 3c/° with an isoluminant red/green chromaticity. PsychoPy will perform all the necessary spatial and chromatic calculations for the user. Although other packages, such as PsychToolbox, may provide scripts that help the user with these sorts of manipulations, none has such a simple automated approach allowing the user to refer to their stimuli directly in real-world units (Fig. 1).

To aid in experimental control a number of functions are also provided to deal with common designs such as staircase procedures or methods of constant stimuli. For example, the user can create a ‘handler’ for a staircase procedure with various settings such as the desired step size(s) and start point for the staircase. On each trial the handler will automatically provide the next intensity level for the stimulus in the staircase based on the previous response of the participant (for complete example of such an experiment see Tutorial 1 on the project website). These handlers can also help to store information about the experiment, such as stimulus parameters, the pseudo-random order of the stimuli, etc.

Finally, a series of functions are provided to aid in data analysis, such as fitting of curves and the use of statistical resampling methods. For example, the following example script (Code Snippet 1) takes a list of intensity values and the mean responses at those intensities. The PsychoPy class FitWeibull then fits a psychometric function to the data. From this we can retrieve the parameters of the fit, request the value of the function at some given intensity values, or the inverse at some given response value(s).

**Figure:**
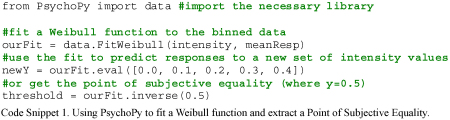

### 3.2 Ease of use

The syntax of the Python language and the PsychoPy library is remarkably easy to understand, especially to anyone with experience in object-oriented languages. As an example, Code Snippet 2 generates a window in which it draws a Gabor of 50% contrast whose grating drifts at a rate of 3 Hz.

As with any language the particular syntax takes time to learn but Python has numerous advantages over Matlab^®^. Most notable is the fact that it was built from scratch as an object-oriented language. For the developer this makes it easier to use and reuse code. For the user, the code remains readable and it becomes extremely easy to identify what functions apply to what types of object. In Code Snippet 2 a clock object is created, which has the method getTime() associated with it. Under the object-oriented programming model the user can create as many clock objects as they choose each of which can return its own (different) time. Another advantage to Python is the ability to name the arguments given to a function with unused arguments being given some default value. In the code below the Window object has numerous additional arguments not shown here (to control the color of the window, whether it appears full screen, etc.) but these were not needed in this instance. The argument naming feature means that we didn’t need to specify all the arguments in their correct order up to the argument that we did actually wish to use (in this case units = “deg”). There are many additional features to the language, like the ability to concatenate strings directly (“Hell” + “o” = “Hello”) or the wide range of available data types, but these are beyond the scope of the current paper. Many additional demo programs including screenshots are available on the project website (www.PsychoPy.org).

**Figure:**
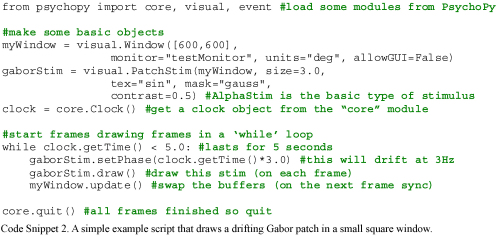

### 3.3 Temporal accuracy and speed

The timing precision is one of the most critical issues for a neuroscience experiment. As you might expect from a platform-independent package, the timing precision of PsychoPy is at least partly dependent on the system clock on which it is running. On most computers this is accurate to the order of microseconds and should have sub-millisecond precision on any modern machine for the purpose of measuring user responses.

For the purposes of synchronizing to a video display, PsychoPy uses a double-buffered display method and the buffer swapping is synchronized to the vertical blank period of the screen, provided that the graphics card in question supports that function (most do). This means that all drawing commands are sent to a hidden copy of the screen and then, when given the command to update, the hidden copy swaps with the currently displayed screen image. When synchronized to the vertical blank, no other commands will be executed until this screen flipping has occurred. Provided that all drawing to the hidden buffer can be completed within the time between vertical blanks (11.7 ms at a refresh rate of 85 Hz) this method provides an extremely robust timing mechanism. The question of whether or not all drawing commands are completed within the necessary time is dependent on the complexity of drawing, the speed of the computer, and its graphics card, but typically the drawing of several hundred moderately complex stimuli is possible. For random-dot stimuli, thousands of simple elements can be rendered within a frame (up to 3500 could be drawn without dropping any frames on a test system with an AMD Athlon 3000+, 512 MB RAM, nVidia GeForceFX 5500). Around 80 Gabor stimuli can be drawn with an updated position/phase/orientation within a single frame. Many more can be drawn if their position is constant. Critically, PsychoPy will optionally test for and report dropped frames throughout the experiment.

For occasions where even more (or more complex) stimuli are necessary, C/C++ extensions can be added to the package to increase the speed of the rendering. For precise synchronization with external hardware, the parallel port and serial port provide very fast methods for sending TTL pulses and characters or other data.

### 3.4 Hardware extensions

PsychoPy has very simple methods to access the parallel and serial ports where available, making interfaces for hardware easy to build. It already provides support for Bits++ (Cambridge Research Systems, Cambridge, UK), Spectrascan PR650 (Photo Research, California, USA), fORP MRI response box (Current Designs Inc., Philadelphia, USA) but should be able to interface with almost anything that uses serial or parallel ports or emulates a computer device such as mouse, keyboard or joystick. In fact, since Python is extensible in C, it should be capable of interfacing with any piece of hardware for which the user has a driver.

To provide the experimenter with greater range of stimulus contrasts than are available using standard graphics cards (with 8 or 10 bit digital–analogue-converters, DACs), PsychoPy integrates with the Bits++ hardware. The Bits++ unit sits between the digital video output of the video card and provides a 14-bit VGA signal out to the monitor. This system provides 14-bit DACS whose outputs are set via lookup-tables that be changed effortlessly every frame (or even within a frame). In its standard mode a lookup table inside the Bits++ unit contains 256 values (corresponding to the output values from the monitor) which correspond to any choice of 14-bit values to be passed to the monitor.^1^ The system is independent of computer platform or of any particular software library and is relatively inexpensive way to produce stimuli with very precise contrast values.

Other devices such as the SpectraScan PR650 can be controlled by the serial port of the computer (or via a USB adaptor that mimics a serial port, where none are available). This is the means by which, for instance, the MonitorCenter instructs the PR650 to make a measurement during automated calibrations and the means by which it retrieves the luminance and/or intensity spectrum after that measurement. The serial port is also one means by which PsychoPy scripts can receive input from the fORP MRI response box allowing the recording of triggers from magnetic resonance imaging (MRI) hardware and responses from MR-compatible button-boxes. The fORP system can simply emulate keyboard presses via the USB port if a serial port is unavailable.

### 3.5 Mechanisms for support

Support for the software is primarily maintained through the website at http://www.psychopy.org. The site is wiki-based, allowing users themselves to directly edit the pages and contribute to or correct documentation. There is also a mailing list through which the users can get support from the original author and also provide support for peers. PsychoPy's code itself is simple, transparent and included with the software so that it too can be modified and improved by the users themselves.

## 4 Discussion

The ideal software package for visual neuroscience should be based on free libraries, open-source (so that the scientist can determine exactly what is happening behind the scenes), simple to use, capable of generating stimuli on-the-fly (rather than from pre-computed movies), and be platform independent (at least for Windows^®^, Macintosh, and Linux environments). The package should also be readily extensible, to handle new technologies and hardware as they are available. Although a number of stimulus presentation packages are already available to neuroscientists (for example, see the list compiled by Strasburger, 2005), none fulfilled all of the above criteria at the time of writing. Table 1 shows a comparison of the features of three such packages in current use and development.

Presentation^®^ (Neurobehavioural Systems Inc, www.neurobs.com) is a commercial package that is available strictly on Windows^®^, is not free and comes without source code. It is also not designed to construct stimuli itself but to render movies and images that have been pre-made in some other package. Of the free software, Psychtoolbox (Brainard, 1997; Pelli, 1997a,b) is the most mature, being used by a very large number of labs worldwide. While Psychtoolbox has been an invaluable tool to many vision scientists (and cognitive neuroscientists) it is built on top of Matlab^®^, which is expensive and comes without source code.

One package with a similar ethos and underlying mechanics to PsychoPy is The Vision Egg (www.visionegg.org) by Andrew Straw. This library, designed originally to study the visual system of the fly is a very powerful package built on top of Python and OpenGL. For a good programmer, Vision Egg achieves its goals very well, providing a powerful and highly optimized system for visual stimulus presentation and interactions with hardware (including the ability to run experiments remotely across a network). Straw does, however, adhere very strongly to an object-oriented model of programming which can be harder for relatively inexperienced programmers, like most scientists, to understand. For instance, the temporal control of experiments in Vision Egg is predominantly though the use of presentation loops, whereby the user sets an object to run for a given length of time, attaches stimuli to it, attaches it to a screen and then tells it to ‘go’. In contrast PsychoPy uses object-oriented programming only where objects make intuitive sense outside the realms of a programming language. For instance, the notion that stimuli or windows are types of object is quite intuitive. For controlling the way in which stimuli appear during a trial, on the other hand, PsychoPy allows the user to create a simple sequence of events (e.g. draw fixation point, wait for 200 ms, draw stimulus, wait for key press, etc.), which is hopefully more intuitive than a presentation object to which events are attached.

PsychoPy aims to provide scientists with an easy and intuitive way to generate experimental control programs, combining the power and freedom of Python/OpenGL with the ease of use found in PsychToolbox. It already contains a wide variety of tools allowing everything from the generation of stimuli and presentation protocols to the logging and analysis of data. With a growing community and continued active development it will only get better.

## Figures and Tables

**Table 1:**
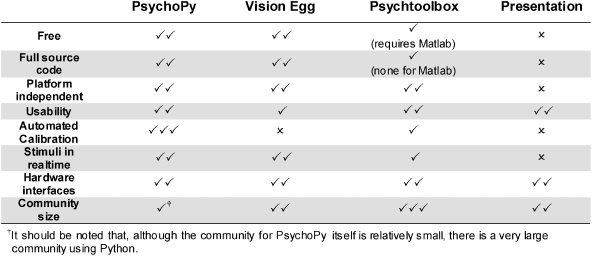
A summary table of features for several frequently used software packages

For further details see main text.

## Footnotes

1 Bits++ has additional modes which can present more than 256 concurrent RGB values, but which sacrifice colour information (Mono++) or screen resolution (Colour++).
